# Plectrabarbene, a New Abietane Diterpene from *Plectranthus barbatus* Aerial Parts

**DOI:** 10.3390/molecules25102365

**Published:** 2020-05-20

**Authors:** Nawal M. Al Musayeib, Musarat Amina, Gadah Abdulaziz Al-Hamoud, Gamal A. Mohamed, Sabrin R.M. Ibrahim, Samah Shabana

**Affiliations:** 1 Department of Pharmacognosy, Pharmacy College, King Saud University, Riyadh 11451, Saudi Arabia; mamina@ksu.edu.sa (M.A.); galhamoud@ksu.edu.sa (G.A.A.-H.); 2 Department of Natural Products and Alternative Medicine, Faculty of Pharmacy, King Abdulaziz University, Jeddah 21589, Saudi Arabia; gamals2001@yahoo.com; 3 Department of Pharmacognosy, Faculty of Pharmacy, Al-Azhar University, Assiut Branch, Assiut 71524, Egypt; 4 Department of Pharmacognosy, Faculty of Pharmacy, Assiut University, Assiut 71526, Egypt; sabrinshaur@gmail.com; 5 Pharmacognosy Department, Faculty of Pharmaceutical Sciences and Drug Manufacturing, Misr University for Science and Technology (MUST), Giza 12511, Egypt; rssmelhaggar@yahoo.com

**Keywords:** *Plectranthus barbatus*, Labiatae, plectrabarbene, abietane diterpene, acetylcholinesterase inhibition, molecular docking

## Abstract

A new abietane diterpene namely plectrabarbene (**2**), together with two known compounds: sugiol (**1**) and 11,14-dihydroxy-8,11,13-abietatrien-7-one (**3**) have been isolated from the aerial parts of *Plectranthus barbatus* Andr. (Labiatae). The structures of these compounds were determined by various spectral techniques (e.g., UV, IR, NMR, and FAB) and by comparison with the literature data. A molecular docking study of the isolated diterpenes (**1**–**3**) was performed with AChE to gain an insight into their AChE inhibition mechanism. The results of docking experiments revealed that the all tested compounds showed binding affinity at the active site of AchE in comparison to donepezil.

## 1. Introduction

**Figure 1:**
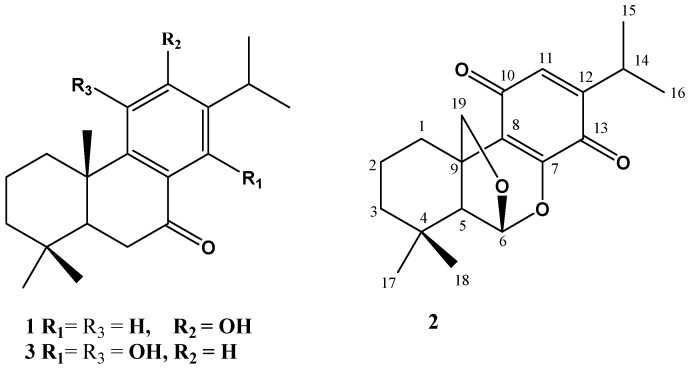
Chemical structure of compounds **1–3**.

The genus *Plectranthus* is constituted of around 350 species and its distribution is restricted to tropical and subtropical regions of Asia, Africa, and Australia [1,2]. The species of *Plectranthus* are known as producers of diterpenoids, flavonoids, phenolic constituents, and essential oils [3]. *Plectranthus barbatus* Andr. is one of the most popular medicinal plant in the genus *Plectranthus* and possesses various potential biological activities [4,5]. *P. barbatus* is native to and common throughout tropical India and Africa but well known in the Southeast and Northeast regions of Brazil [6]. Traditionally, *P. barbatus* has been reported for diverse medical uses in Indian Hindu and Ayurvedic medicine as well as in folk medicine in Brazil, China, and Africa [7]. The majority of traditional uses are for stomach ache, intestinal disturbances, heart failure, hypertension, colic, eczema, respiratory problems, central nervous system disorders, and cancer prevention [8,9,10]. Extensive phytochemical investigation of *P. barbatus* has revealed the presence of diterpenes in particular abietane and abietanoid derivatives as the main components [11]. Other classes of compounds isolated from this plant include flavonoids, steroids, and essential oils [6,12]. Earlier, our group has reported the anticancer activity of extracts of aerial parts and isolation of 2`*R*-hydroxydocosanoylursa-12-en-3β-ol (barbaterpene) and 3β,5α-dihydroxy-stigma-7(8),22-diene (barbatusterol) from *P. barbatus* [13,14]. In continuation to our systematic research on bioactive components from *P. barbatus* of Saudi origin, we herein reported the isolation and identification of a new abietane diterpene, plectrabarbene (**2**) and two known compounds identified as sugiol (**1**) and 11,14-dihydroxy-8,11,13-abietatrien-7-one (**3**) based on spectral data (UV, IR, MS, NMR, and MS) and compared with literature values (Figure 1). This paper describes the detailed spectral evidence as well as molecular docking studies of the isolated metabolites.

## 2. Results and Discussion

### 2.1. Chemistry

**Figure 2:**
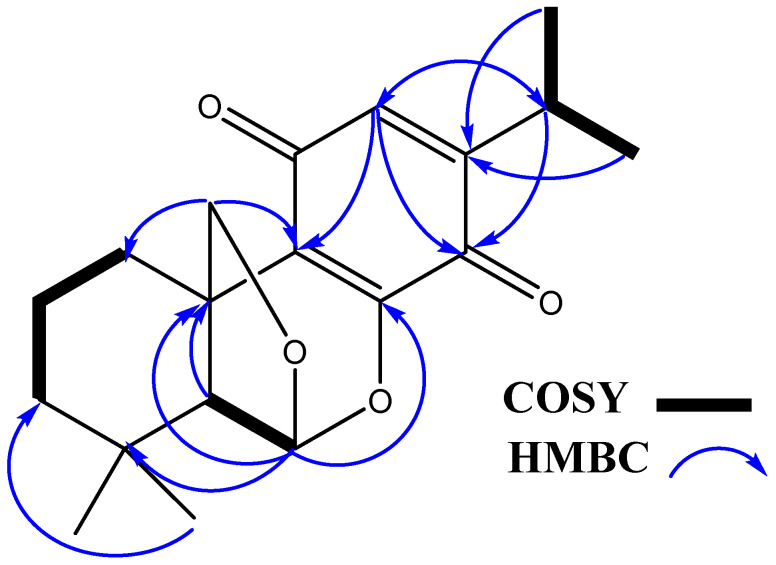
Some Key ^1^H-^1^H COSY and HMBC correlations of **2**.

Compound **2** was isolated from ethyl acetate fraction *P. barbatus* by vacuum liquid chromatography over silica gel. It was crystallized from methanol as yellow glassy needles, m.p. 124–128 °C; [α]_D_^25^: +23.7 (*c* 0.05 MeOH). The FAB mass spectrum of **2** gave a [M + Na]^+^ ion at *m*/*z* 339.47 and [M + H]^+^ ion at *m*/*z* 317.4510 (Figure S6), indicating its molecular mass to be 316 and suggesting its molecular formula to be C_19_H_24_O_4_, in combination with elemental analysis. It had UV absorptions at 365 and 400 nm. The IR spectrum of **2** displayed an absorption band at 1610 cm^−1^ suggesting the presence of C=C olefinic groups and characteristic absorption bands for quinone at 1650 and 1605 cm^−1^ with the absorption at 1650 cm^−1^ being of greater intensity than at 1605 cm^−1^ [15]. The ^1^H NMR spectrum of **2** (Table 1, Figure S1) in CD_3_OD showed proton signals for two tertiary methyl groups at δ 1.11 and 1.02 (each *s*, 3H, H-17, 18), two secondary methyl groups at δ 1.12 (d, 3H, *J* = 7.0 Hz, H-15) and 1.13 (*d*, 3H, *J* = 7.0 Hz, H-16), oxymethylene protons at δ 4.27 (*d*, 1H, *J* = 14.0 Hz, H- 19A) and δ 4.21 (*d*, 1H, *J* = 14.0 Hz, H- 19B), and an aromatic proton at δ 6.32 (*s*, 1H, H-11). These signals correlated with the C-atom signals at δ 32.4 (C-17), 21.1 (C-18), 20.2 (C-15), 20.2 (C-16), 80.9 (C-19), and 131.0 (C-11), respectively in the HSQC spectrum (Figures S2 and S4) indicating that **2** was an abietane type diterpene derivative containing a *p*-benzoquinone C-ring [16]. This was established by observed ^1^H-^1^H COSY cross-peaks and further confirmed by the HMBC cross peaks of H-14/C-12, C-13, C-15, and C-16, H-15 and H-16/C-12 and C-14, H-17/C-4, C-5, and C-18, H-18/C-3, C-4, C-5, and C-17, and H-19/C-1, C-5, C-6, C-7, C-8, and C-9 (Figure 2). In the ^13^C NMR spectrum of (**2**), a total of 19 carbon signals was recorded in CPD (complete proton decoupled) spectrum. In DEPT 45 and DEPT 135, a total of 12 carbons were recorded. The DEPT, HSQC, and HMBC spectra (Figures S3–S5) indicated the presence of four methyls, four methylenes, four methines, and seven quaternary carbons. The ^13^C NMR showed C-atom signals attributed to C-10 and C-13 quinone carbonyls at δ 186.0 and 181.5, respectively. The abietane skeleton of **2** was confirmed by ^13^C NMR spectrum, which showed oxymethylene and oxymethine carbon signals at δ 80.9 and 102.6 suggested that these two carbon atoms should be attached to an ether moiety. This was further supported by the broad oxymethine singlet at δ 5.74 (H-6) and by the two doublets obtained at δ 4.27 and 4.21 assignable to the protons of oxymethylene group (H-19). The difference in chemical shift between oxymethylene and oxymethine groups more than 1.0 ppm proved that the latter two groups were found in different environments, and suggested that one hydrogen points towards and other away from the aromatic ring, which is in agreement with the ether being located at C-19. The oxymethine signal at δ 5.74 exhibited HMBC correlation with the peaks at δ 80.9 (C-19), 42.5 (C-9), and 29.8 (C-4), suggesting the existence of an ether bridge between C-6 and C-19, forming a tetrahydrofuran ring. This was further supported by the HMBC correlations methylene signals at δ 4.27 and 4.21 to C-1 (δ 24.9) and C-9 (δ 42.5). The relative configuration of H-6 was observed to be α-orientated as per the Dreiding model, indicating that the six-membered rings in abietane derivative linked from C-19 to C-6 [17]. The chemical structure of **2** was further supported by mass spectrum, which revealed the fragment peaks at *m*/*z* 287.0267 [M−CH_2_O]^+^, 273.1288 [M−CHO]^+^, 259.1366 [M−CH_2_]^+^, and 245.1361 [M−CH_3_]^+^, which is dominated by an ion peak at *m*/*z* 287 due to the removal of CH_2_O fragment from C-9 position of the molecular ion, a typical base peak in abietatriene compounds (Figure S7) [18]. Thus, the structure of **2** was unambiguously elucidated as depicted and the trivial name plectrabarbene was given to it (Figure 1).

The known compounds were identified as sugiol (**1**) and 11,14-dihydroxy-8,11,13-abietatrien-7-one (**3**) by comparing their spectral and physical data with the literature [19,20].

### 2.2. Molecular Docking of Isolated Compounds

The biggest challenge faced by the pharmaceutical industry is to ensure the availability of new drugs in the market. The number of new drugs produced, approved, and released each year remains steady, despite the constant rise in funds for research and developments [21]. This situation has inspired researchers to develop different strategies for the identification of new lead compounds [22], as the high price of biological assay and methodologies have restricted their use [23]. Furthermore, difficulties arise when the active constituent occurs in low quantities, which means large amounts of natural products are needed to isolate the component of interest [24]. Keeping in consideration the availability of several potential biological targets for new drugs, a recent docking-based virtual screening (DBVS) approach plays an important role in the identification of promising bioactive constituents. It is a theoretical-based approach that facilitates the characterization of lead components from the three-dimensional structure of the receptor of interest using docking programs. These docking programs estimate the affinity of a ligand (small molecule) for a specific molecular target to measure the interaction energy of the resulting innovative complex. Moreover, starting from the complex between the ligand and the receptor, visualization software can present the intermolecular interaction that is responsible for molecular recognition. Thus DBVS can identify the most promising lead compounds for biological assays and decrease the costs associated with drug development [22,25].

**Figure 3:**
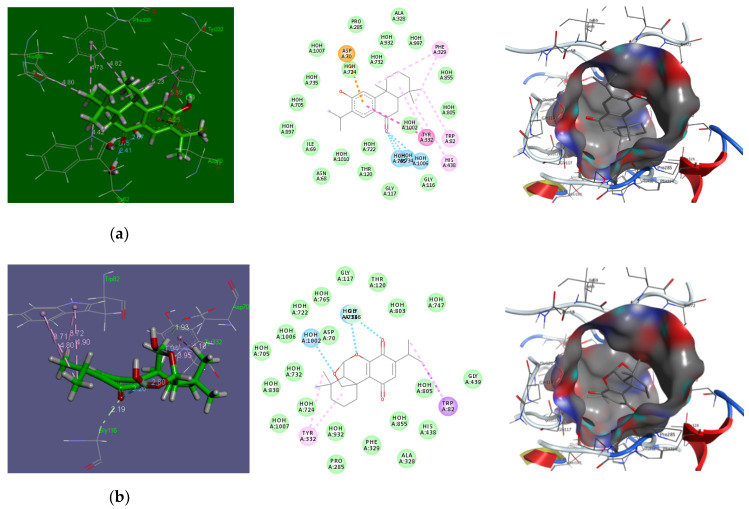

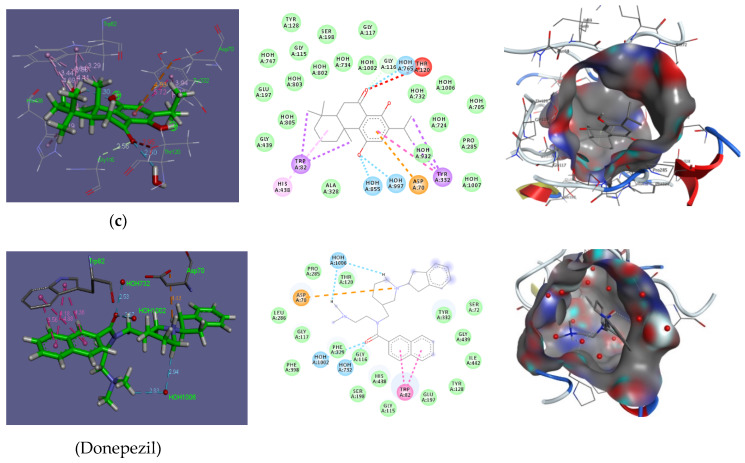
Docking of compounds **1** (**a**)**, 2** (**b**)**,** and **3** (**c**) in Acetylcholine esterase enzyme in comparison to donepezil as a reference drug.

The molecular docking of the isolated diterpenes (**1–3**) was performed with AChE to gain an insight into their mechanism of AChE inhibition. From the results of docking experiments, it was found that all the tested compounds showed a binding affinity at the active site of AchE comparison to donepezil (Table 2 and Figure 3).

According to the docking models, compound **2** interacted with AChE by forming two carbon hydrogen bonds with ASP70 and GLY116, three conventional hydrogen bonds with water molecules H_2_O-734 and H_2_O-1002, and several pi-alkyl interactions with amino acids TRP82 and TYR332 (Table 2). Other amino acid residues such as THR120, PHE329, HIS438, and GLY439 also interact with AChE and stabilize the AChE-compound **2** complex (Figure 3b). The compound (**2**)–AChE complex was stabilized by −4.7 kcal mol^−1^ of binding energy (Table 2). Similarly, compound (**1**) attached to AChE via one pi-Anion interaction with ASP70, three hydrogen bonds with water molecules H_2_O-734, H_2_O-756, and H_2_O-1006, and several pi-alkyl interactions with amino acids TRP82, PHE329, TYR332, and HIS438 (Table 2). Other amino acid residues involved in stabilizing the compound **1**–AChE complex were ILE69, GLY116, THR120, PRO285, and ALA328 (Figure 3a). The binding energy of a compound **1** and AChE complex was −6.3 kcal mol^−1^ (Table 2). Furthermore, compound **3** attached to AChE through one pi-Anion interaction with ASP70, two hydrogen bonds with water molecules, H_2_O-756 and H_2_O-855, Pi–Pi interaction with TYR332, and several pi-alkyl interactions with amino acids TRP82 and TYR332 (Table 2). Other amino acid residues involved in stabilizing the compound **3**–AChE complex were GLY116, GLU197, PRO285, ALA328, and GLY439 (Figure 3c). The binding energy of the compound **3** and AChE complex was −2.65 kcal mol^−1^ (Table 2).

Our results indicated that compound **2**, interacts with the key residues of AChE such as ASP70 (carbon hydrogen bonds), H_2_O-1002 (hydrogen bonding), and TRP82 (pi–alkyl interactions). Likewise, compound **1** interacts with some key residues of AChE through pi–Anion interaction (ASP70), hydrogen bonding (H_2_O-734, H_2_O-756, and H_2_O-1006), and several pi-alkyl interactions (TRP82, PHE329, TYR332, and HIS438). Similarly, compound **3** interacts with some key residues of AChE through pi–Anion interaction (ASP70), hydrogen bonding (H_2_O-756 and H_2_O-855), and pi-alkyl interactions (TRP82 and TYR332). The results of the interaction between donepezil (reference drug) and AchE were tabulated in (Table 2) and illustrated in (Figure 3). Thus, the results of the docking experiments revealed that all the tested diterpenes showed a strong binding affinity at the active site of AchE when compared to donepezil, suggesting that these compounds could be future promising drugs for the treatment of Alzheimer’s. 

### 2.3. Possible Biosynthetic Pathway of Compound **2**

**Figure 4:**
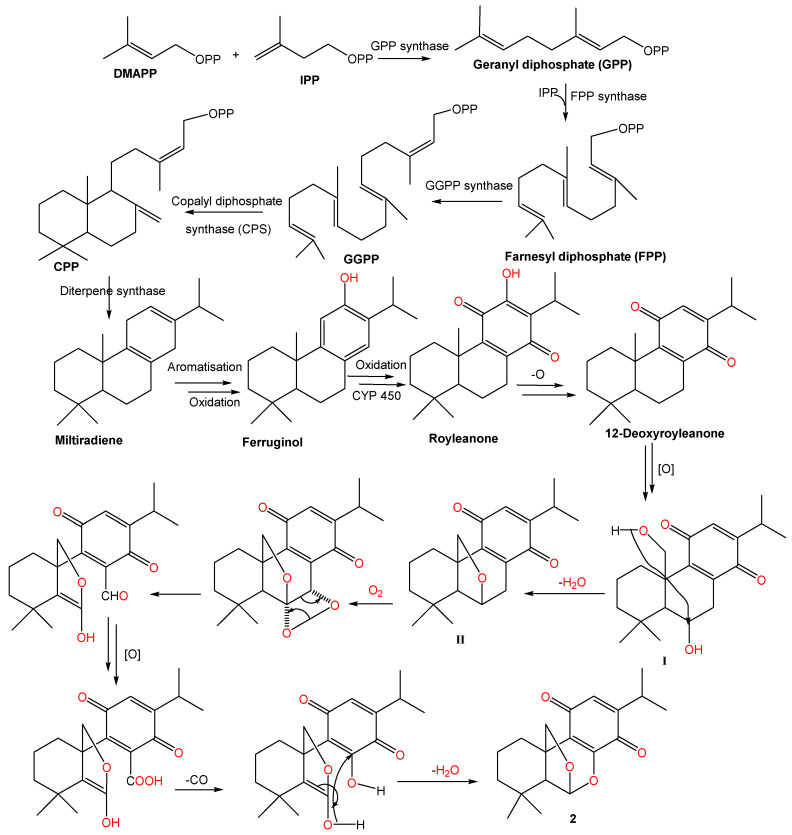
Biogenetic pathway proposed for plectrabarbene (**2**).

The 20 carbon atom skeleton of labdane diterpenes, is synthesized from geranylgeranyl diphosphate (GGPP), which is formed through sequential head-to-tail condensation of isopentenyl diphosphate (IPP) and dimethylallyl diphosphate (DMAPP) [26,27]. Copalyl diphosphate synthase (CPS) catalyzes the bicyclization of GGPP to copalyl diphosphate (CPP), and followed by the production of an intermediate miltiradiene, which through spontaneous aromatization and oxidation converted to ferruginol [28,29]. Further reactions such as hydroxylation and oxygenation, which are catalyzed by cytochrome P450 enzymes are followed by quinone formation in the C-ring of 12-deoxyroyleanone [30]. Subsequent hydroxylation at C-6 and oxidation at C-20 (**I**), followed by ether formation would then result in the formation of an intermediate (**II**) [30]. Moreover, subsequent reactions take place leading to the formation of **2** [30] (Figure 4). 

## 3. Materials and Methods

### 3.1. General

All spectral data were obtained on various instruments. The Buchi apparatus model B-545 was used to record the melting point and was uncorrected. Optical rotations were taken on the PerkinElmer model 341 LC polarimeter (Perkin-Elmer Inc, Massachusetts, MA, USA). The IR and UV spectra were measured on the JASCO 320-A and a Hitachi-UV-3200 spectrophotometers (Kyoto, Japan), respectively. The NMR spectral analyses were obtained by the Bruker Avance DRX 700 MHz spectrometer (Rheinstetten, Germany), in either CDCl_3_ or CD_3_OD. FAB-MS and EI-MS were determined by using the JEOL SX 102/DA-6000 and Agilent 6320 ion trap mass spectrometers (ThermoFinnigan, Bremen, Germany), respectively. Column and gel permeation chromatographic separations were performed on silica gel 60 (Merck, 0.04–0.063 mm, Darmstaddt, Germany) and sephadex LH-20, respectively. TLC analyses were carried on pre-coated SiO_2_ DC-Plastikfolien 60 F_254_ plates with detection accomplished by spraying with CeSO_4_, I_2_, and vanillin-H_2_SO_4_ followed by heating at 100 °C. The molecular docking studies were conducted using Auto Dock Vina, M.G.L tools 1.5.7, and Discovery Studio 4.5 as a visualizer. The human-acetylcholinesterase enzyme (AChE) (PDB 6O4W) was used as a receptor for the docking study and donepezil as a reference drug.

### 3.2. Plant Material

*P. barbatus* Andr. aerial parts were collected from its natural habitat of Al-Taif, Saudi Arabia in March 2014 and identified by Dr. M. Yousef, a taxonomist at the Department of Pharmacognosy, College of Pharmacy, King Saud University, Riyadh province where the voucher specimen (15732) was deposited in the herbarium.

### 3.3. Extraction and Isolation

Two kilograms of the air-dried powder of *P. barbatus* aerial parts were extracted four times with 70% of EtOH (4 × 2.5 L) at room temperature. The resulting organic extracts were pooled, filtered through Whatman paper no. 1, and concentrated under reduced pressure to yield 58.4 g of the dark brown residue. The later was suspended in a water/methanol mixture and partitioned successively with *n*-hexane, CHCl_3_, EtOAc, and *n*-BuOH to obtain *n*-hexane (9.5 g), CHCl_3_ (13.5 g), EtOAc (17.4 g), and *n*-BuOH (13.2 g) soluble fractions. The EtOAc soluble fraction was applied on a vacuum liquid chromatography column (VLC) packed with silica gel (230–400 mesh, Merck, Germany) and eluted in an increasing polarity manner with a CHCl_3_/MeOH mixture to afford four sub-fractions (Pb1 to Pb4). Subfraction Pb1 (2 g) was chromatographed over silica gel column chromatography (SiO_2_ CC) (50 × 2 cm × 100 g) eluted in gradient *n*-hexane/EtOAc to give **1** (5.4 mg, colorless crystals). Repeated column chromatography of combined subfractions Pb2 and Pb3 (8.5 g) over SiO_2_ CC using CH_2_Cl_2_/MeOH and further purification over sephadex LH-20 using MeOH as an eluent afforded **2** (7.3 mg, yellow glassy needles). Further column chromatography of subfraction Pb4 (4.2 g) on SiO_2_ using CHCl_3_/MeOH gradient yielded **3** (6.3 mg, yellow needles).

Compound **1**: yellow needles, m.p. 280–282 °C; [α]_D_^25^: +27.9 (*c* 1.0 CHCl_3_); IR (KBr) γ*_max_*: 3127, 2765, 1645, 1578, 1565, 1462, 1372, 1340 cm^−1^; ^1^H NMR (CDCl_3_, 700 MHz): δ 0.95 (3H, s, H-18), 1.01 (3H, *s*, H-19), 1.25 (3H, s, H-20), 1.28 (3H, d, *J* = 7.5, 10 Hz, H-16), 1.27 (3H, d, *J* = 7.5 Hz, H-17), 1.88 (dd, *J* = 3.5 Hz, H-5), 2.71 (1H, dd, *J* = 4.5 Hz, H-6α), 2.62 (1H, dd, *J* = 4.5 Hz, H-6β), 3.16 (1H, m, H-15), 6.72 (1H, s, H-11), 7.94 (1H, s, H-14); EI-MS: *m*/*z* 300 (calcd. for C_20_H_28_O_2_).

Compound **2**: yellow glassy needles, m.p. 128 °C; [α]_D_^25^: +23.7 (*c* 0.05 MeOH); UV(MeOH) λ*_max_* (log *ε*): 365 (3.45), 400 (3.12) *nm*; IR (KBr) γ*_max_*: 2965, 1610, 1650, 1605 cm^−1^; NMR (CD_3_OD, 700 and 176 MHz): see Table 1. FABMS *m*/*z*: 339.47 [M + Na]^+^, 317.45 [M + H]^+^ (calcd. for C_19_H_24_O_4_).

Compound **3**: colorless cyrstals, m.p. 178–180 °C; [α]_D_^28^: +58.3 (*c* 0.05 MeOH); IR (KBr) γ*_max_*: 3253, 2567, 1640, 1572, 1545, 1458, 1365, 1334 cm^−1^; ^1^H NMR (CDCl_3_, 700 MHz): δ 0.98 (3H, s, H-18), 1.00 (3H, *s*, H-19), 1.41 (3H, s, H-20), 1.21 (3H, d, *J* = 7.0 Hz, H-16), 1.23 (3H, d, *J* = 7.0 Hz, H-17), 3.33 (1H, sept, H-15), 1.86 (1H, dd, *J* = 9.6, 7.2 Hz, H-5), 2.67 (1H, dd, *J* = 14.0, 7.2 Hz, H-6 α), 2.69 (1H, dd, *J* = 14.0, 9.6 Hz, H-6 α); EI-MS: *m*/*z* 316 (calcd. for C_20_H_28_O_3_).

### 3.4. Molecular Docking Studies

The molecular docking studies were conducted using Auto Dock Vina, M.G.L tools 1.5.7, and Discovery Studio 4.5 as a visualizer. The human-acetylcholinesterase enzyme (AChE) (PDB 6O4W) was used as a receptor for the docking studies and donepezil as a reference drug. The validation of the docking accuracy was investigated to ensure a valid docking and to evaluate the effect of the water molecules. The co-crystalized ligand in the acetylcholinesterase enzyme was docked to its corresponding protein (in the presence and in the absence of water molecules) and the RMSD values between the co-crystalized ligand and the docked pose were calculated. The obtained success rates of AutoDock were excellent where the active site of the acetylcholinesterase enzyme has been determined from the binding of a co-crystalized ligand. The energy minimized acetylcholinesterase enzyme, the co-crystalized ligand and the three isolated compounds were finally prepared in the right format using MGL tools 1.5.7 for conducting the docking study by Auto Dock Vina that requires both the receptor and the ligands in pdbqt format [31]. The grid was generated for the protein using MGL tools 1.5.7. Auto Dock Vina achieves an approximate two orders of magnitude speedup compared to the molecular docking software Auto Dock 4, while also significantly improving the accuracy of the binding mode predictions. Further speedup is achieved from parallelism, using multithreading on multi-core machines. Auto Dock Vina uses the Auto Dock score that calculates free binding energies and the iterated local search global optimization algorithm [32,33,34]. The result of docking was visually inspected by Discovery Studio 4.5 visualizer. The evaluation of candidates was based on binding affinity and interaction with receptor.

## 4. Conclusions

Three pure compounds (**1**–**3**) were isolated and identified from the aerial parts of *P. barbatus*; one of them is a new natural chemical entity (**2**). Structures of the isolated compounds were characterized on the basis of various spectroscopic analyses. In addition, molecular docking of these isolated compounds was carried out with AChE and all the compounds showed strong binding affinity at the active site of AchE.

## Figures and Tables

**Table 1:** NMR spectral data of compound **2** (CD_3_OD, 700, and 176 MHz).

No.	δ_H_ [mult., *J* (Hz)]	δ_C_ (mult.)	HMBC
**1**	2.74 brd (14.0) 1.62 m	24.9 CH_2_	2, 3, 5, 9, 19
**2**	1.66 m	18.0 CH_2_	9, 19
**3**	1.50 brd (14.0) 1.33 m	38.8 CH_2_	1, 2, 4, 5, 18
**4**	-	29.8 C	-
**5**	2.10 brs	53.3 CH	6, 8, 9, 17, 18, 19
**6**	5.74 brs	102.6 CH	4, 5, 7, 8, 9, 19
**7**	-	128.4 C	-
**8**	-	152.1 C	-
**9**	-	42.5 C	-
**10**	-	186.0 C	-
**11**	6.32 s	131.0 CH	7, 8, 9, 12, 13, 14, 15, 16
**12**	-	150.8 C	-
**13**	-	181.5 C	-
**14**	2.97 m	26.1 CH	11, 12, 13, 15, 16
**15**	1.12 d (7.0)	20.2 CH_3_	12, 14
**16**	1.13 d (7.0)	20.2 CH_3_	12, 14
**17**	1.11 s	32.4 CH_3_	4, 5, 18
**18**	1.02 s	21.1 CH_3_	3, 4, 5, 17
**19**	4.27 d (14.0) 4.21 d (14.0)	80.9 CH_2_	1, 5, 6, 7, 8, 9

**Table 2:** Molecular docking parameters of the interaction between isolated diterpenes (**1**–**3**) and AChE in comparison to donepezil as a reference drug.

Name of Bond and Amino Acid Involved in Interaction	Type of Interaction	Distance (Å)	Binding Energy (kcal mol^−1^)
**Compound (1)**			
ASP70: OD2-drug TRP82-drug C18 PHE329-drug C19 PHE329-drug C20 TYR332-drug TYR332-drug HIS438-drug C19 H_2_O 734:H2-drug O1 H_2_O 765:H1-drug O1 H_2_O1006:H1-drug O1	Pi-Anion interaction Pi-Alkyl interaction Pi-Alkyl interaction Pi-Alkyl interaction Pi-Pi interaction Pi-Alkyl interaction Pi-Alkyl interaction Hydrogen bond Hydrogen bond Hydrogen bond	4.25 4.42 4.73 4.82 4.80 5.69 5.23 2.41 2.15 2.67	−6.3
**Compound (2)**			
ASP70: OD2- drug H16 TRP82- drug C18 TRP82- drug C18 TRP82- drug C19 TRP82- drug C19 GLY116: HA1- drug O4 TYR332- drug C19 TYR332-drug H_2_O 734: H2-drug O2 H_2_O 734: H2-drug O1 H_2_O 1002: H2- drug O4	Carbon hydrogen bond Pi-Alkyl interaction Pi-Alkyl interaction Pi-Alkyl interaction Pi-Alkyl interaction Carbon hydrogen bond Pi-Alkyl interaction Pi-Alkyl interaction Hydrogen bond Hydrogen bond Hydrogen bond	1.93 3.71 3.72 4.90 4.80 2.19 4.10 3.95 2.26 2.8 1.94	−4.7
**Compound (3)**			
ASP70: OD2- drug TRP82-drug C18 TRP82-drug C18 TRP82-drug TRP82-drug TRP82-drug C20 TRP82-drug C20 TYR332-drug C16 TYR332-drug C1 7TYR332-drug GLY116: HA1-drug O3 THR120: OG1-drugO3 H_2_O 855:H2-drug O1 H_2_O 765:H1-drug O3	Pi-Anion interaction Pi-Alkyl interaction Pi-Alkyl interaction Pi-Alkyl interaction Pi-Alkyl interaction Pi-Alkyl interaction Pi-Alkyl interaction Pi-Alkyl interaction Pi-Alkyl interaction Pi-Pi interaction Carbon hydrogen bond Un favorable bond Hydrogen bond Hydrogen bond	4.93 3.44 3.58 4.69 4.41 3.29 4.64 3.38 3.94 5.72 2.56 2.95 2.30 2.60	−2.6
**Donepezil (Reference)**			
H_2_O 732: O-drug OAY H_2_O 1002: O-drug OAY H_2_O 1006: O-drug HAJ1 H_2_O 1006: O-drug HAV2 ASP70: OD1-drug: NAK TRP82-drug TRP82-drug TRP82-drug TRP82-drug	Hydrogen bond Hydrogen bond Hydrogen bond Hydrogen bond Ionic bond Pi-Pi Interaction Pi-Pi Interaction Pi-Pi Interaction Pi-Pi Interaction	2.53 2.6 72.94 2.83 4.43 3.65 4.18 4.36 4.88	−7.32

## Supplementary Materials

The following are available online at https://www.mdpi.com/1420-3049/25/10/2365/s1, Figure S1: ^1^H NMR spectrum of compound **2** (CD_3_OD, 700 MHz), Figure S2: ^13^C NMR spectrum of compound **2** (CD_3_OD, 176 MHz), Figure S3: DEPT spectrum of compound **2**, Figure S4: HSQC spectrum of compound **2**, Figure S5: HMBC spectrum of compound **2**, Figure S6: HR-ESI-MS spectrum of compound **2**, Figure S7: MS spectrum of compound 2.
