# Supplementary material for: Plectrabarbene, a New Abietane Diterpene from Plectranthus barbatus Aerial Parts
Source: Molecules. 2020 May 20;25(10):2365. doi: 10.3390/molecules25102365 (PMC7288077; doi:10.3390/molecules25102365)
Supplement: Supplementary file 1 [file molecules-25-02365-s001.pdf]

molecules

Supplementary materials

Plectrabarbene, a New Abietane Diterpene from *Plectranthus barbatus* Aerial Parts

Nawal M. Al Musayeib [1,*], Musarat Amina[1], Gadah Abdulaziz Al-Hamoud [1], Gamal A. Mohamed [2,3], Sabrin R.M. Ibrahim [4] and Samah Shabana [5]

[1] Department of Pharmacognosy, Pharmacy College, King Saud University, Riyadh 11451, Saudi Arabia; mamina@ksu.edu.sa (M.A.); galhamoud@ ksu.edu.sa (G.A.A.-H.)
[2] Department of Natural Products and Alternative Medicine, Faculty of Pharmacy, King Abdulaziz University, Jeddah 21589, Saudi Arabia; gamals2001@yahoo.com
[3] Department of Pharmacognosy, Faculty of Pharmacy, Al-Azhar University, Assiut Branch, Assiut 71524, Egypt
[4] Department of Pharmacognosy, Faculty of Pharmacy, Assiut University, Assiut 71526, Egypt; sabrinshaur@gmail.com
[5] Faculty of pharmaceutical sciences and drug manufacturing, Pharmacognosy Department, Misr University for Science and Technology (MUST), 6 October, Egypt; rssmelhaggar@yahoo.com
* Correspondence: nalmusayeib@ksu.edu.sa

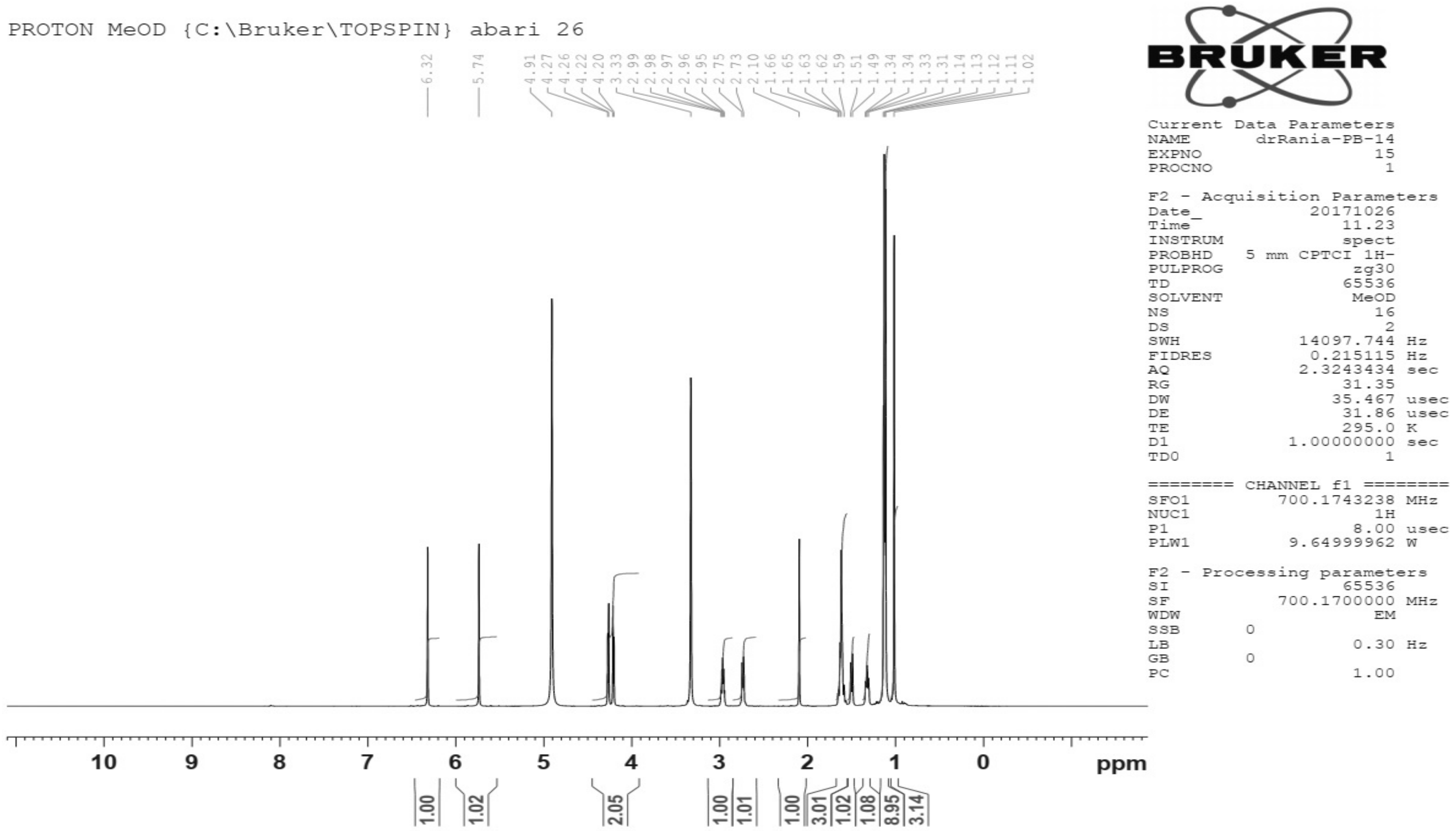

Figure S1: ^{1}H NMR spectrum of compound **2** (CD_3OD, 700 MHz).

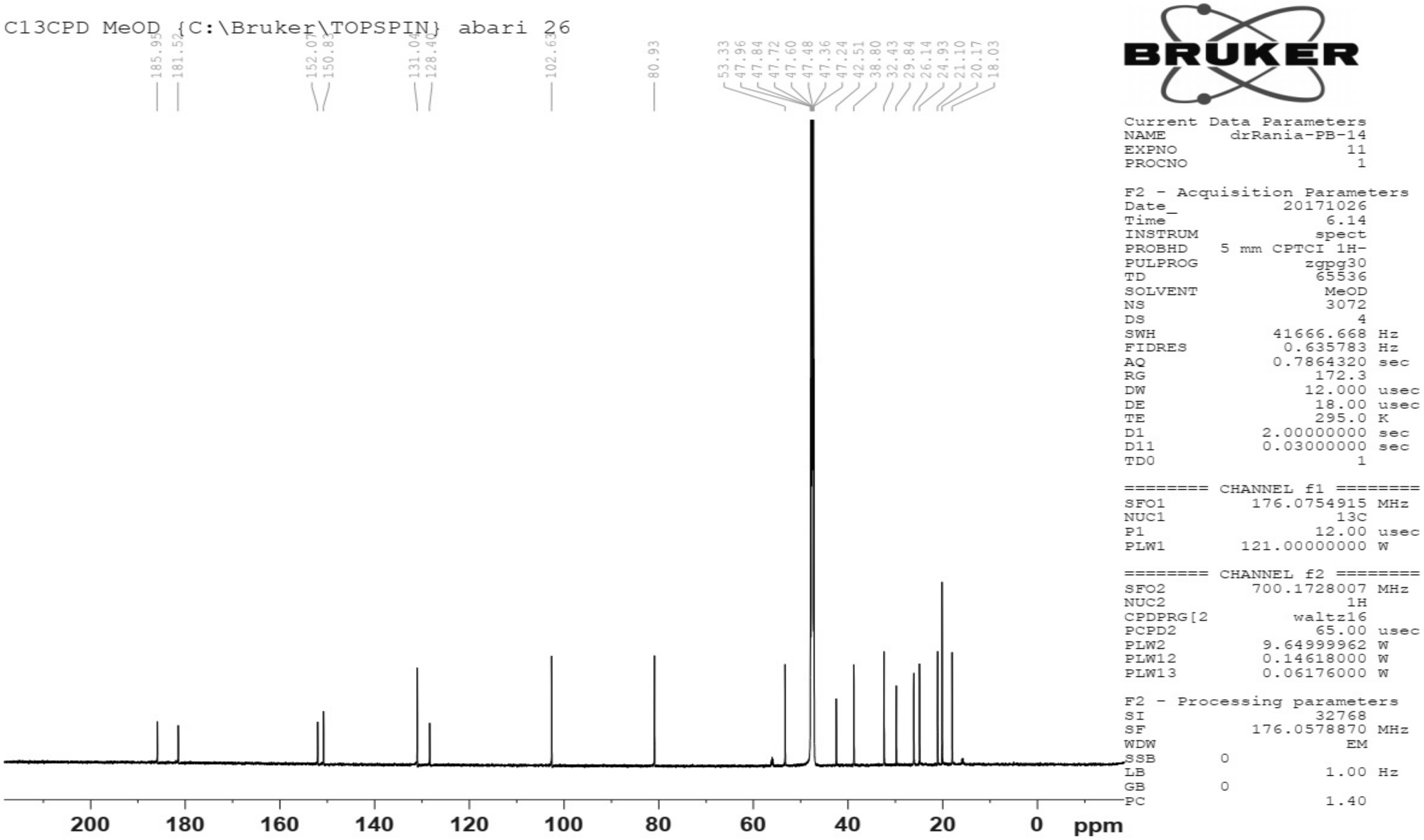

Figure S2: ^{13}C NMR spectrum of compound **2** (CD_3OD, 176 MHz).

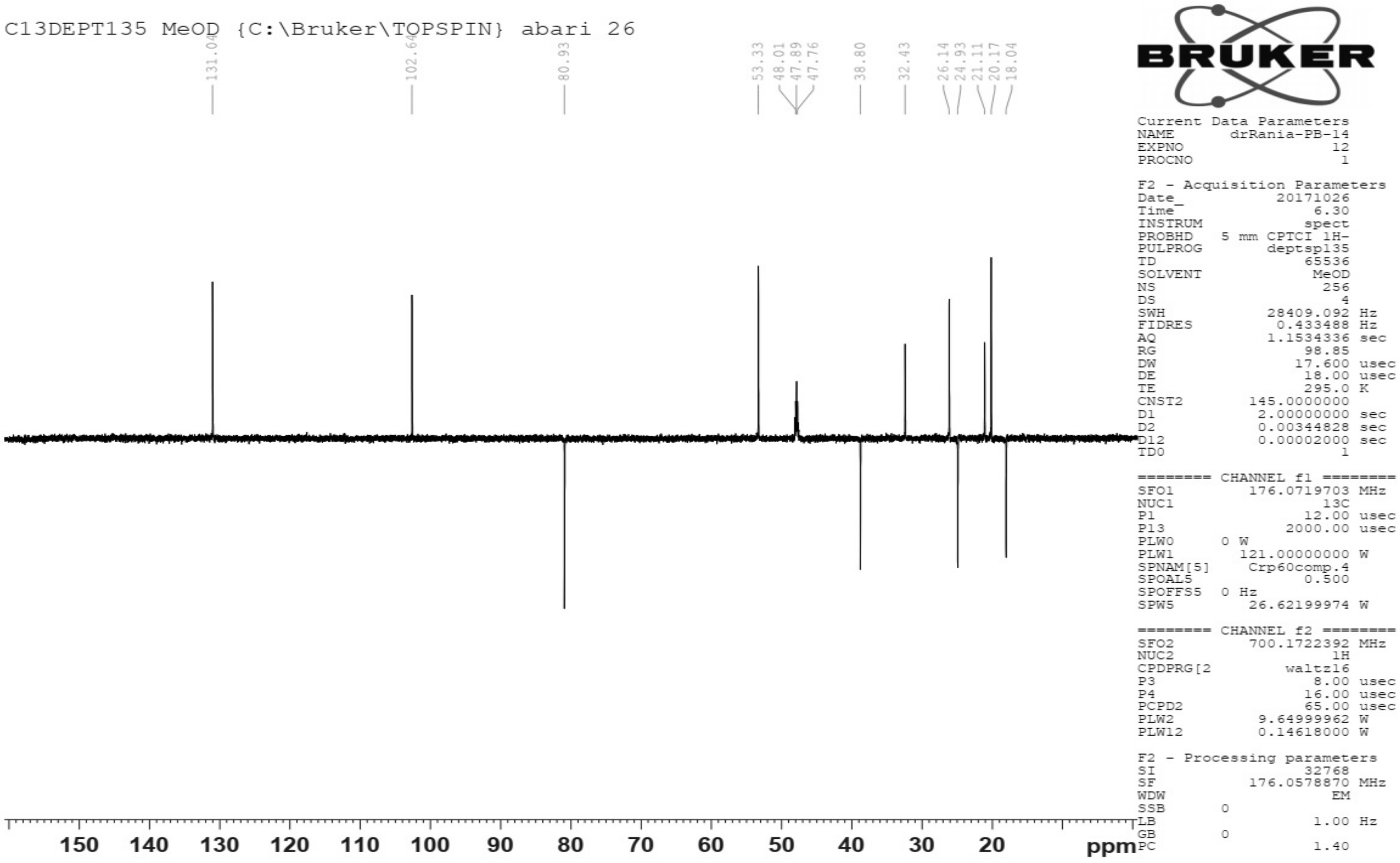

Figure S3: DEPT spectrum of compound **2.**

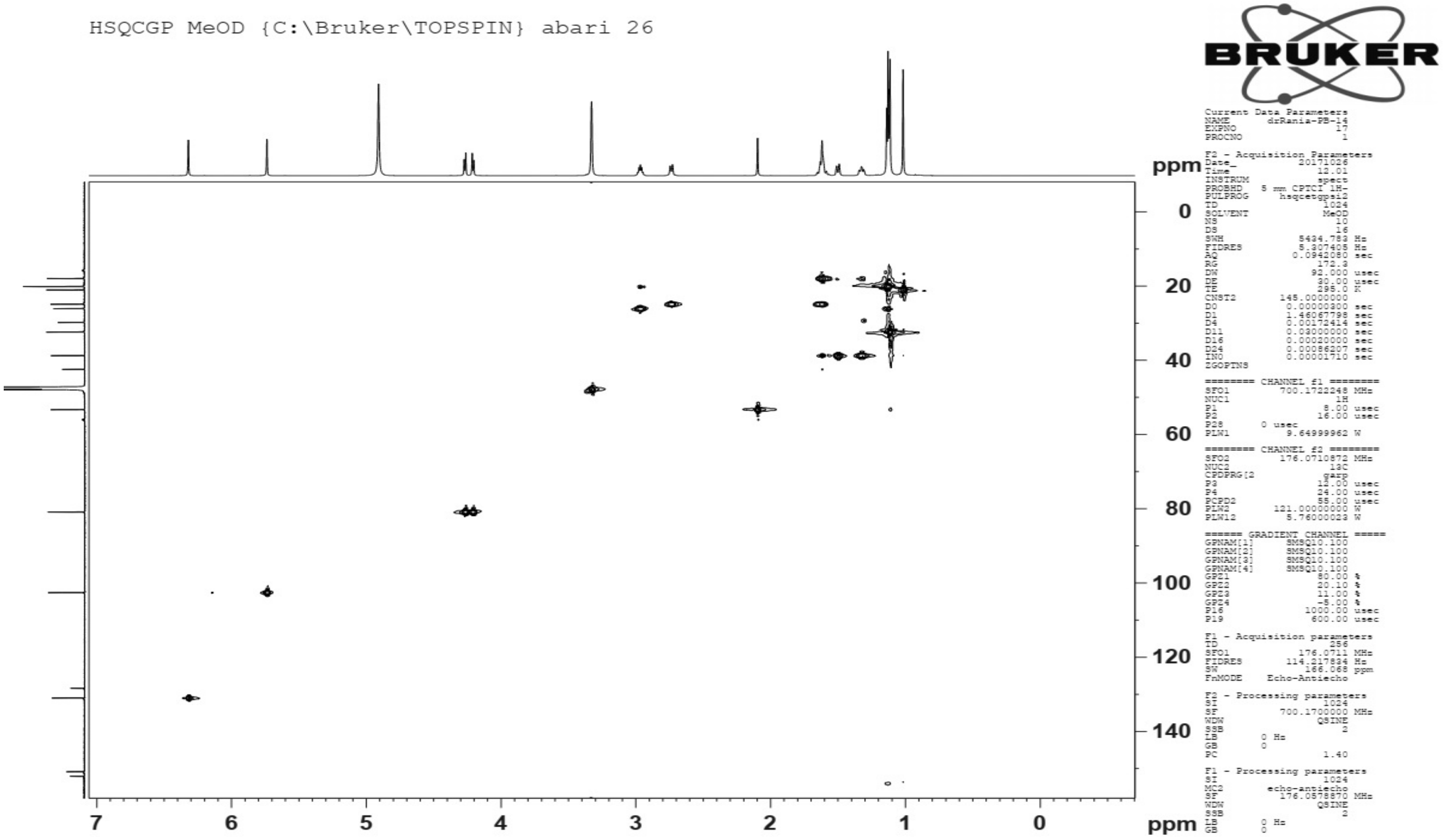

Figure S4: HSQC spectrum of compound **2.**

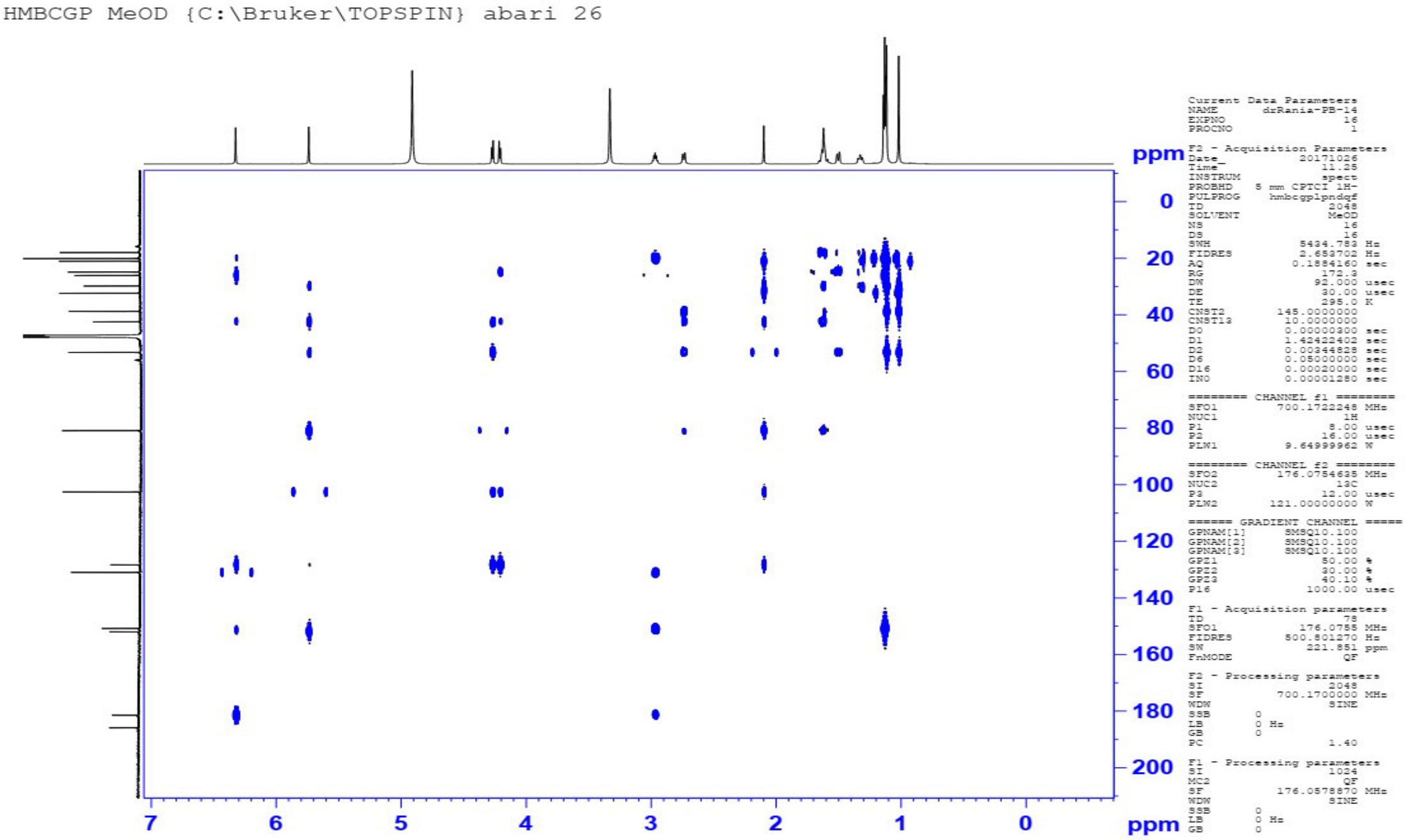

Figure S5: HMBC spectrum of compound **2.**

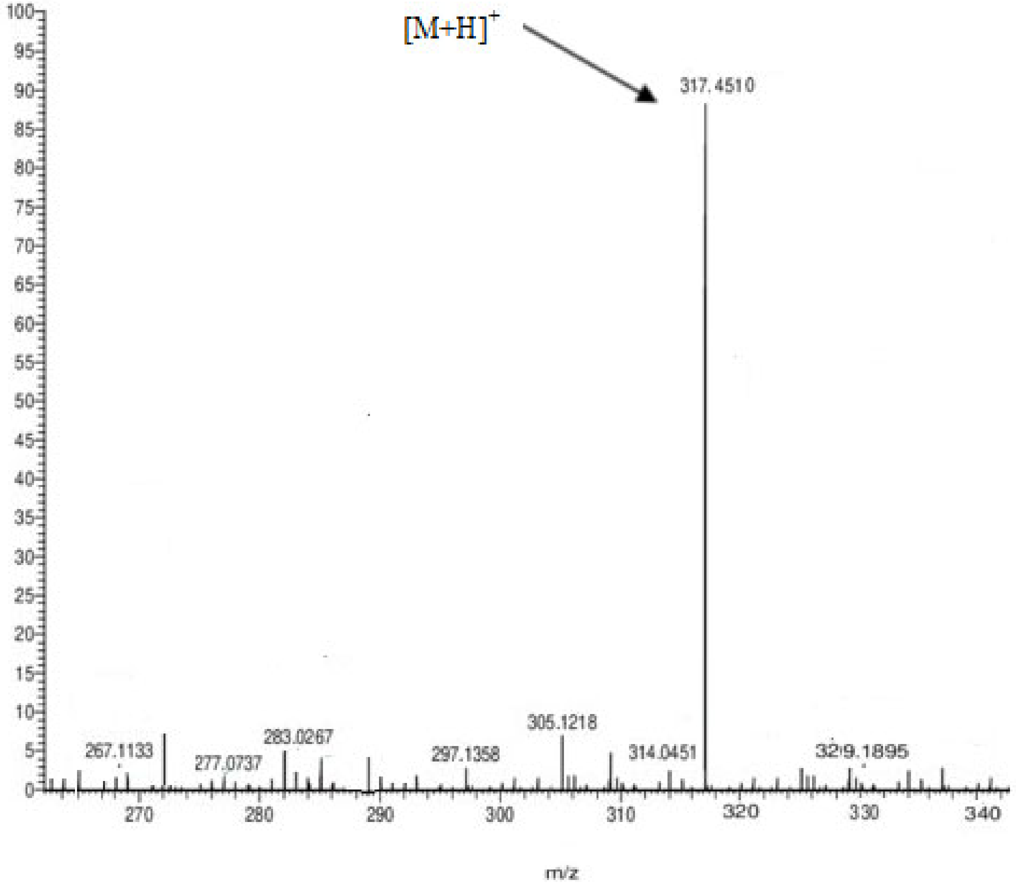

Figure S6: HR-ESI-MS spectrum of compound **2.**

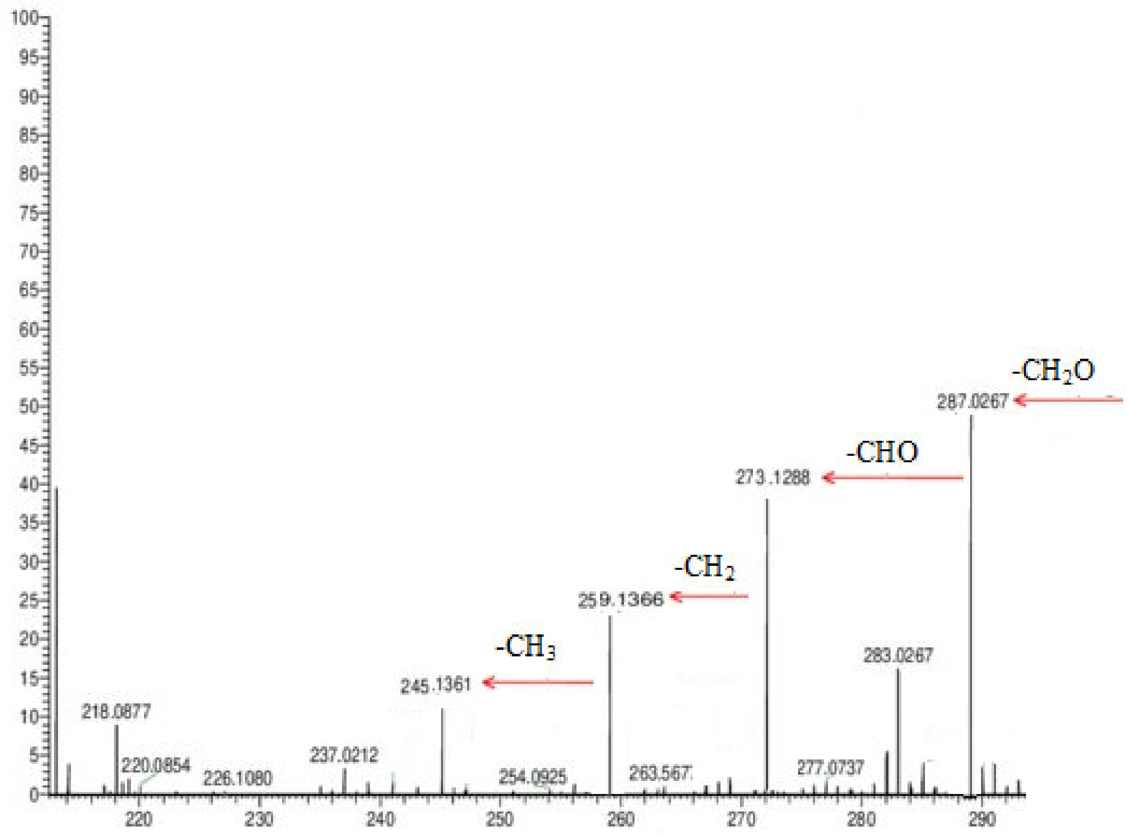

Figure S7: MS spectrum of compound **2.**
